# Wave Reflection and Ventriculo-Arterial Coupling in Bicuspid Aortic Valve Patients With Repaired Aortic Coarctation

**DOI:** 10.3389/fped.2021.770754

**Published:** 2022-01-28

**Authors:** Elena Giulia Milano, Sandra Neumann, Froso Sophocleous, Giulia Pontecorboli, Stephanie L. Curtis, Radwa Bedair, Massimo Caputo, Giovanni Battista Luciani, Chiara Bucciarelli-Ducci, Giovanni Biglino

**Affiliations:** ^1^Bristol Heart Institute, University Hospitals Bristol & Weston, NHS Foundation Trust, Bristol, United Kingdom; ^2^Department of Surgery, Dentistry, Pediatrics and Gynecology, University of Verona, Verona, Italy; ^3^Bristol Medical School, University of Bristol, Bristol, United Kingdom; ^4^Cardiovascular and Thoracic Department, Careggi University Hospital, Florence, Italy; ^5^Royal Brompton and Harefield Clinical Group, Guy's and St Thomas' NHS Foundation Trust, London, United Kingdom; ^6^National Heart and Lung Institute, Imperial College London, London, United Kingdom

**Keywords:** aortic coarctation, bicuspid aortic valve, congenital heart disease, wave intensity analysis, cardiac magnetic resonance, ventriculo-arterial coupling, ventricular strain

## Abstract

**Background:**

Ventriculo-arterial (VA) coupling in bicuspid aortic valve (BAV) patients can be affected by the global aortopathy characterizing BAV disease and the presence of concomitant congenital lesions such as aortic coarctation (COA). This study aimed to isolate the COA variable and use cardiovascular magnetic resonance (CMR) imaging to perform wave intensity analysis non-invasively to shed light on VA coupling changes in BAV. The primary hypothesis was that BAV patients with COA exhibit unfavorable VA coupling, and the secondary hypothesis was that BAV patients with COA exhibit increased wave speed as a marker of reduced aortic distensibility despite successful surgical correction.

**Methods:**

Patients were retrospectively identified from a CMR database and divided into two groups: isolated BAV and BAV associated with repaired COA. Aortic and ventricular dimensions, global longitudinal strain (GLS), and ascending aortic flow data and area were collected and used to derive wave intensity from CMR data. The main variables for the analysis included all wave magnitudes (forward compression/expansion waves, FCW and FEW, respectively, and reflected backward compression wave, BCW) and wave speed.

**Results:**

In the comparison of patients with isolated BAV and those with BAV associated with repaired COA (*n* = 25 in each group), no differences were observed in left ventricular ejection fraction, GLS, or ventricular volumes, whilst significant increases in FCW and FEW magnitude were noted in the BAV and repaired COA group. The FCW inversely correlated with age and aortic size. Whilst the BCW was not significantly different compared with that in patients with/without COA, its magnitude tends to increase with a lower COA index. Patients with repaired COA exhibited higher wave speed velocity. Aortic wave speed (inversely related to distensibility) was not significantly different between the two groups.

**Conclusion:**

In the absence of a significant restenosis, VA coupling in patients with BAV and COA is not negatively affected compared to patients with isolated BAV. A reduction in the magnitude of the early systolic FCW was observed in patients who were older and with larger aortic diameters.

## Introduction

Changes in ventriculo-arterial (VA) coupling are a key determinant of ventricular energetics (1). Considering the arterial side of the VA equation, both global and local changes can result in alterations of coupling efficiency. These changes can include overall alterations in arterial stiffness of the vessel as well as local changes in the vessel anatomy such as focal stenoses or sites of bifurcations. In this light, patients with bicuspid aortic valve (BAV) represent an interesting population in which to assess VA coupling, considering the presence of both global vascular abnormalities (BAV aortopathy) and the presence of concomitantly associated congenital lesions such as aortic coarctation (COA).

Wave intensity analysis (2) is an established method to measure the interaction between the ventricle and the remainder of the vasculature, and it has been used in other congenital heart diseases such as hypoplastic left-heart syndrome as well as aortic COA. In particular, previous studies exploring the hemodynamics of COA using wave intensity analysis observed that its presence results in a substantial backward reflection wave, in turn associated with left ventricular (LV) hypertrophy (3) and unfavorable LV energetics.

This demonstrates how the analysis of VA coupling can highlight the relationship between changes on the arterial side and the function of the LV and their possible repercussions on ventricular remodeling.

Importantly, wave intensity analysis lends itself to both invasive and non-invasive formulations, the latter making the analysis compatible with routinely acquired medical imaging data.

In this study, wave intensity analysis was performed non-invasively from cardiovascular magnetic resonance (CMR) data. This study focuses specifically on the assessment of the presence of a repaired COA in a population of patients with BAV, trying to isolate the COA variable from the BAV-associated aortopathy. The hypothesis underpinning the study is that BAV patients with COA exhibit unfavorable VA coupling. A secondary hypothesis is that BAV patients with COA also exhibit increased wave speed as a marker of reduced arterial distensibility.

The aim is to explore the difference in VA coupling between patients with isolated BAV and patients with BAV and repaired COA.

## Methods

### Patient Population

Patients for this study were retrospectively identified from a database of 525 clinical CMR scans in patients with BAV acquired at the Bristol Heart Institute between 2011 and 2016 (4).

Ethical approval was not required by the local Research and Innovation Department in light of the retrospective nature of the study.

Patients were excluded based on the following criteria: (i) previous surgery on the aortic valve and/or surgery of the aortic root or ascending aorta, (ii) associated congenital heart defects apart from repaired aortic COA, (iii) undefined aortic valve morphology, (iv) presence of aortic valve stenosis (any degree), (v) presence of moderate to severe aortic regurgitation, (vi) presence of severe reCOA (COA index >0.5), and (vii) suboptimal image quality.

Twenty-five patients with BAV and repaired COA and 25 patients with isolated BAV were ultimately included from the above-mentioned sample.

### CMR Imaging

All scans were acquired at 1.5 T (Avanto, Siemens Healthineers, Erlangen, Germany). Demographic and clinical information were gathered from CMR reports, together with aortic dimensions, valvular anatomy, presence of COA, presence and severity of valve dysfunction, LV volumes, LV mass, and LV ejection fraction (LVEF).

Aortic regurgitation was graded, according to regurgitant fraction quantification, as mild (<30%), moderate (31–49%), and severe (>50%). Aortic stenosis was classified, according to valve planimetry, as mild (>1.5 cm^2^), moderate (1.0–1.5 cm^2^), and severe (<1 cm^2^) (5).

Severity of the reCOA was defined based on a COA index (6), i.e., a ratio of the aortic diameter at the level of the isthmus over the diameter of the descending aorta at the level of the diaphragm. Severity of reCOA was thus graded as absent (COA index >0.85), mild–moderate (COA index = 0.5–0.85), or severe (COA index <0.5).

Global longitudinal strain (GLS) was measured with the dedicated tool in the CVI42 software (Circle Cardiovascular Imaging, Calgary, Canada) by manually contouring the endocardial and epicardial borders of three long axis projections (four-chamber, three-chamber, and two-chamber views of the LV) in end-systole and end-diastole and propagating the regions of interest (ROIs) semiautomatically throughout the cardiac cycle, followed by manual correction.

**Figure 1:**
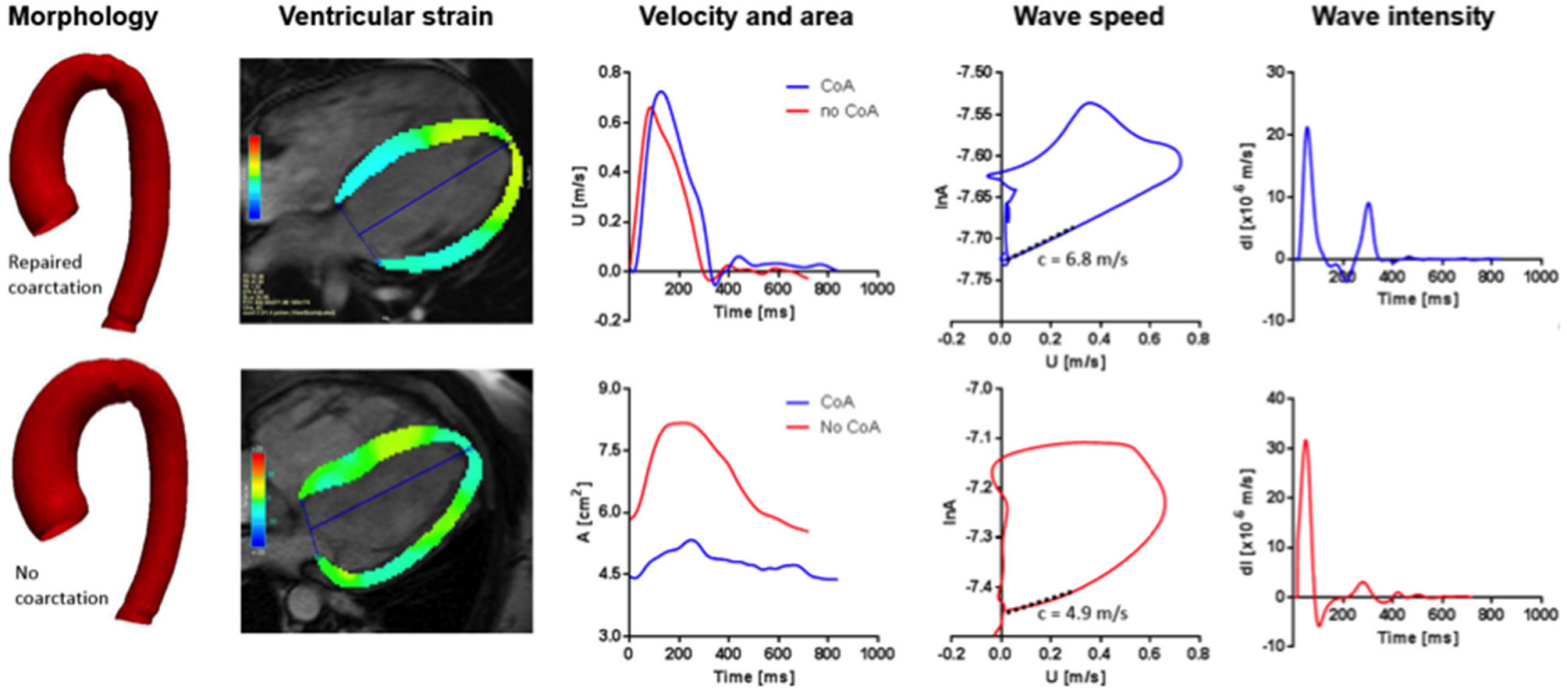
From left to right: Aortic arch template of patients with BAV and repaired aortic COA (upper) and isolated BAV (lower); four-chamber view for GLS analysis in the diastole (upper) and systole (lower); velocity (upper) and area (lower) curves derived from phase-contrast analysis at the level of the ascending aorta; wave speed in patients with repaired COA (upper) and isolated BAV (lower); examples of wave intensity analysis patterns in a patient with repaired COA (upper) and isolated BAV (lower).

The phase-contrast (PCMR) flow sequences acquired in the proximal ascending aorta at the level of the left pulmonary artery were selected for wave intensity analysis (7). Image segmentation was performed using the Flow package of CVI42 (Circle Cardiovascular Imaging), contouring the area of the aorta and propagating the ROI semiautomatically throughout the cardiac cycle, obtaining the corresponding flow velocity and area curves (Figure 1). The latter were used to derive fractional changes in area (dlnA) and velocity differentials (dU) to calculate wave speed and perform wave intensity analysis.

### Aortic Distensibility

Processing of the aortic velocity (U) and area (A) signals and subsequent calculations were all performed in Matlab (MathWorks, Natick, MA, USA) using an in-house script, including steps of interpolation and resampling of the signals to 1 ms. Changes in U and A are related through the water hammer equation:


(1) $$\begin{array}{r} \text{d}{\text{U}}_{\pm }=\pm \text{c:mtext> :mtext>d:mtext>l:mtext>n}{\text{A}}_{\pm } \end{array}$$

where the + and – subscripts indicate the forward-traveling and the backward-traveling components of the wavefront, respectively.

Similar to the pressure–velocity loop method (8), the U and lnA signals were plotted against each other, and the slope of the early systolic linear portion of the loop yields an estimate of wave speed (c). This approach, mathematically based on the Riemann method of characteristics, is based on the observation that in early systole the relationship between pressure and velocity is generally linear, with the slope of the first portion of the loop being proportional to c (9). Knowledge of c, in turn, allows direct estimation of aortic distensibility (D) based on the Bramwell–Hill equation (10):


(2) $$\begin{array}{r} \text{D}=1/\text{ρ}{\text{c}}^{2} \end{array}$$

where ρ is the density of blood.

### Wave Intensity Analysis

Wave intensity is a haemodynamic index that describes the working condition of the heart in relation to the remainder of the vasculature (3, 9), defined as the product of simultaneous pressure and velocity differentials at a given point in the circulation:


(3) $$\begin{array}{r} \left(\text{d:mtext>P}/\text{d:mtext>t}\right)\times \left(\text{d:mtext>U}/\text{d:mtext>t}\right) \end{array}$$

where dP/dt and dU/dt represent changes in pressure and velocity measured simultaneously at a given location. As an analytical tool, wave intensity offers insights into haemodynamic changes and wave propagation, as it quantifies the intensity and energy carried by waves traveling in the blood (both forward, i.e., away from the heart, and backward, i.e., toward the heart) in the time domain (11). The clinical meaning of changes in wave intensity has been discussed extensively in the relevant literature, particularly indicating that the magnitude of the first positive peak of the aortic wave intensity pattern positively correlates with ventricular dP/dt and the second positive peak in late systole negatively correlates with the diastolic time constant τ (12). The first peak (a forward-traveling compression wave, FCW) can thus be considered an indicator of contractile performance of the ventricle, whilst the second peak (a forward-traveling expansion wave, FEW) can be considered an indicator of isovolumic relaxation. Wave intensity thus carries information on both contractility and protodiastolic relaxation of the left ventricle (12, 13), and it has been shown to hold prognostic value, predicting cardiovascular events independently of other cardiovascular risk factors or being independently associated with cognitive decline (14, 15).

### Data Analysis

Statistical analysis was carried out in Stata (v 13.1, StataCorp, College Station, TX, USA). Continuous variables are reported as mean ± SD, when normally distributed, and median (IQR) when not normally distributed, based on visual assessment of the data. Differences between continuous variables were assessed with either a Student's *t*-test or Mann–Whitney test, as appropriate. Categorical variables are reported as proportions or percentages, and differences between categorical variables were assessed with chi-square test. Associations between clinical, anatomical, and functional variables were investigated using univariate linear regression analysis. If multiple predictors were found to be significantly associated with an outcome of interest, these were further tested in a multivariable regression model. A *p* < 0.05 was taken to indicate statistical significance.

## Results

Demographic characteristics of the study population, together with anatomical and functional data, are summarized in Table 1. There were no statistically significant differences in age (at the time of the CMR scan), sex, or BSA amongst the two groups.

**Table 1:** Demographic characteristic, anatomical, and functional data.

	**BAV + repaired COA**	**Isolated BAV**	***p*-value**
Age (years)	30.7 (21.7–34.9)	33.5 (26.9–50.0)	0.09
Male sex (%)	68	48	0.25
BSA	1.8 (1.7–2.0)	1.9 (1.7–2.2)	0.10
BAV–RL fusion pattern (%)	88	76	0.47
Presence of AR (%)	36	52	0.39
Peak AoV velocity (m/s)	1.2 (1.1–1.7)	1.4 (1.2–1.6)	0.95
Forward flow (ml)	82 (71.5–92.5)	85 (74–99)	0.50
Net forward flow (ml)	75 (69.0–90.5)	75 (66.5–92.5)	0.98
Cardiac output (l/min)	5.2 (4.3–6.4)	5.6 (4.8–6.4)	0.38
AoV regurgitant fraction (%)	2 (1.0–6.5)	6 (3.5–11.5)	0.01^*^
SOV (mm/m^2^)	17.3 (15.6–18.9)	20.3 (17.5–21.9)	0.001^*^
AA (mm/m^2^)	14.6 (12.1–18.9)	20.8 (17.7–22.4)	<0.001^*^
COA index	0.73 (0.6–0.8)	–	
Transverse arch (mm)	16 (14.0–18.0)	22 (19.5–24.0)	<0.001^*^
Descending aorta—mid (mm)	19 (16.0–22.0)	21 (19.5–22.5)	0.19
Descending aorta—diaphragm (mm)	16.5 ± 2.7	18.1 ± 2.2	0.024^*^
Arch hypoplasia index	1.0 ± 0.2	1.2 ± 0.2	0.006^*^

*AA, ascending aorta; AoV, aortic valve; AR, aortic regurgitation; BSA, body surface area; SOV, sinus of Valsalva*.

* *p < 0.05*.

Patients with BAV and repaired COA presented overall with smaller diameters at the sinus of Valsalva compared to patients with isolated BAV (17.3 vs. 20.3 mm/m^2^, *p* = 0.001), ascending aorta (14.6 vs. 20.8 mm/m^2^, *p* < 0.001), and the transverse aortic arch (16 vs. 22 mm, *p* < 0.001).

The majority of patients with BAV and repaired COA underwent a surgical repair (92%), whereas only 8% of the patients underwent and interventional repair with stenting. Surgical procedures included end-to-end anastomosis (*n* = 11), subclavian flap aortoplasty (*n* = 2), and unknown (*n* = 10). None of the patients had a significant reCOA (median COA index = 0.73).

Patients with isolated BAV had a higher degree of aortic regurgitation compared to those with repaired COA (regurgitant fraction 2 vs. 6%, *p* = 0.01).

No differences were observed in LVEF and GLS between the two groups, as reported in Table 2. Indexed end-diastolic and end-systolic volumes also did not differ significantly between the two groups.

**Table 2:** Results from CMR analysis.

	**BAV + repaired COA**	**BAV**	***p*-value**
LVEDVi (ml/m^2^)	74 (66–95)	81 (76–97)	0.23
LVESVi (ml/m^2^)	33 (28–37)	30 (23.0–38.5)	0.36
LVEF (%)	61 (57–66)	62 (57.5–69.5)	0.28
GLS (%)	−19 (−20.8 to −16.7)	−18.7 (−21.2 to 16.7)	0.75

*LVEDVi, left ventricular end diastolic volume indexed; LVESVi, left ventricular end systolic volume indexed; LVEF, left ventricular ejection fraction; GLS, global longitudinal strain*.

An example of the results is illustrated in Figure 1. Significant increases in FCW and FEW magnitudes were noted in the repaired COA subgroup, as reported in Table 3. Furthermore, wave intensity revealed the presence of a backward compression wave (BCW), although it was not significantly different when comparing the BAV-with-repaired-COA group with the isolated-BAV group.

**Table 3:** Wave intensity analysis results for the BAV patients with and without COA (BAV + COA vs. isolated BAV).

	**BAV + repaired COA**	**Isolated BAV**	***p*-value**
c (wave speed) m/s	5.9 (3.3–7.8)	4.2 (2.5–6.3)	0.08
D (distensibility) 1/mmHg × 10^−3^	0.0035 (0.002–0.111)	0.0068 (0.003–0.019)	0.08
FCW (m/s) × 10^−5^	2.8 ± 2.0	1.7 ± 1.6	0.04*
FEW (m/s) × 10^−5^	0.42 ± 0.30	0.2 ± 0.2	0.02*
BCW (m/s) × 10^−5^	−0.3 ± 0.5	−0.2 ± 0.1	0.10
FCW/FEW	8.5 ± 5.7	10.4 ± 9.0	0.50
FCW/BCW	15.4 ± 15.6	23.6 ± 38.4	0.60

*FCW, forward compression wave; FEW, forward expansion wave; BCW, backward compression wave*.

Linear regression analysis, in BAV patients with repaired COA, revealed that FCW was inversely associated with age (rho = −0.49, *p* = 0.037) and with increasing diameters at the level of the ascending aortic (rho = −0.625, *p* = 0.001), aortic root (rho = −0.55, *p* = 0.005), and descending aorta (rho = −0.44, *p* = 0.034). It was also noted that the FEW magnitude decreased as aortic diameters increased at the ascending aortic and aortic root (rho = −0.55, *p* = 0.008 and rho = −0.0441, *p* = 0.04, respectively). The BCW in patients with BAV and repaired COA increased in magnitude with worsening of the COA index (rho = −0.435, *p* = 0.043).

Aortic wave speed was higher in the group of BAV with repaired COA (suggesting a degree of reduction of aortic distensibility in these patients), but this difference did not reach statistical significance (c = 5.9 vs. 4.2 m/s, *p* = 0.08).

A ratio of “VA efficiency,” measured as the FCW/FEW ratio, was not different between the two groups (8.5 ± 5.7 vs. 10.4 ± 9.0, *p* = 0.5), as well as the ratio FCW/BCW, both reported in Table 3.

## Discussion

This study examined properties of the aortic arch in a population of BAV patients, assessing the effect of repaired COA on aortic hemodynamics. The main observations from this study were that patients with BAV and repaired COA without any significant reCOA exhibit VA efficiency comparable to those with isolated BAV, as shown by the wave intensity analysis results, and that the presence of residual narrowing, albeit not clinically significant based on the COA index, appeared to be associated with an increment in the magnitude of backward reflected waves. This study also complements and adds to the overall wave intensity literature, particularly because it provides information on patients with BAV only, whereas the literature has generally focused on the presence of the COA, its haemodynamic effect, and different types of COA repair (16, 17).

The study analyzed VA coupling based on the hypothesis that patients with repaired COA would exhibit a reduction in the wave intensity values, reflecting an unfavorable VA coupling scenario, possibly due to anatomical and functional abnormalities.

As far as the aortic arch morphology is concerned, in our population, patients with COA had smaller aortic diameters compared to isolated-BAV patients, with lower diameters at the level of the root and in the ascending aorta, with a degree of transverse arch hypoplasia (4, 18).

With regard to LV functional parameters, there were no differences in global LV systolic function, assessed with LVEF and GLS. Likewise, a difference in VA efficiency was not observed between the two groups. Compared with other studies, ours did not show a correlation between LV deformation parameters and vascular indices (19). However, patients with BAV and repaired COA presented higher values of wave speed (and thus reduced aortic distensibility), compared to isolated-BAV patients, suggesting an increase in aortic stiffness in the first group, albeit results did not reach statistical significance.

A prior larger study employing wave intensity analysis in a population of COA patients reported a significant increase in ascending aortic stiffness compared to healthy controls (2). These results are not confirmed in our population, and this is possibly due to the fact that both our groups, being affected by BAV and related aortopathy, are carriers of structural abnormalities of the aortic wall.

Insight into post-operative COA physiology, especially through exercise studies, highlighted different features, at times conflicting (20), and different factors beyond the aortic anatomy itself have been suggested to be at play, including increased systemic arterial resistance and a hyperdynamic state which can lead to increased systolic blood pressure (20, 21).

The presence of an important BCW has already been described in repaired COA patients (3). Our results confirm the presence of a backward wave in patients with BAV and repaired COA, which was not significantly different in magnitude from that measured in patients with isolated BAV. The presence of the BCW derives from the anatomical restriction representing a reflection site or from an increase in aortic stiffness at the site of COA, or a combination of both factors. It should also be considered that, above and beyond local changes at the COA site, the overall aortopathy may play a role in such a population with isolated BAV, explaining the absence of significant differences amongst the two groups. The presence and magnitude of the BCW have also been associated with increasing LV mass in patients with atherosclerosis (22, 23). Furthermore, hypertensive therapy has been suggested to reduce the magnitude of the BCW in the aorta (22).

This analysis indicated a trend toward an increase of the magnitude of the BCW increases as the COA index decreases, even when the reCOA is not clinically significant (median COA index 0.73). Hashimoto et al. demonstrated that in the absence of an anatomical narrowing or in the presence of a mild reCOA, daily systolic blood pressure is independently associated with the COA index (24). The mechanism through which this phenomenon occurs is not entirely understood and may be in part explained by the increased aortic stiffness in these patients, also presenting histological changes and a reduction in smooth muscle cells vs. collagen (25, 26). The magnitude of the BCW may represent an additional tool to stratify these patients and potentially contribute to inform their treatment. However, additional studies are required to verify the relationship between increasing BCW and significant alterations in blood pressure and/or clinical endpoints.

Despite not observing significant alterations in VA coupling, we found that older patients and those with increased aortic diameters (at the level of the aortic root and in the descending aorta) present a reduction of the FCW, as a surrogate for dP/dt. This finding suggests the possible presence of subgroups with unfavorable anatomical and functional characteristics. Also, whilst this study focused on comparing BAV patients with and without repaired COA, comparison with values reported in the literature for healthy controls in a similar age range suggests a substantial difference, which should be explored further in a single prospective study.

The uptake of parameters derived from imaging-based methodologies, such as statistical shape modeling (27) and wave intensity analysis, could potentially represent a contribution of diagnostic value allowing identification of patient subgroups at risk of developing aortic dilatation, LV systolic and diastolic dysfunction, or systemic hypertension and thus requiring more frequent follow-up (28). In particular, future prospective studies should explore the relationship between morphological characteristics of the aortic arch and changes in aortic distensibility with VA coupling and the development of hypertension (at rest and during exercise).

### Limitations

The main limitations of this study are due to its retrospective nature. Data on arterial blood pressure (cuff measurements) at the time of CMR were not available for most patients as well as LV mass. Whilst the main aim of the study was to explore changes in wave intensity parameters comparing BAV patients with and without COA and as such lacked a group of healthy controls, the small sample size of the study limited subgroup comparisons of interest, such as comparing patients with different COA repairs (different surgical approaches and/or stenting). It would also be interesting to explore any relationship between age of COA repair and wave intensity variables. Whilst the study was based on routinely acquired CMR data, high-temporal-resolution PCMR would be desirable in future prospective studies to confirm observations on changes in wave speed.

## Conclusion

In conclusion, this study explored the effect of the presence of repaired aortic COA on VA coupling in a population of patients with BAV. Patients with BAV and repaired COA, in the absence of a significant reCOA, are not negatively affected with regard to VA coupling compared to patients with isolated BAV. On the other hand, the study suggests that further studies assessing the effect of BAV aortopathy on ventricular energetics are warranted, expanding the current BAV literature.

## Data Availability Statement

The raw data supporting the conclusions of this article will be made available by the authors, without undue reservation.

## Author Contributions

GB and CB-D designed the study. EM, FS, and GP collected the data and performed the analysis on CMR images. SN and GB performed the WIA. GB performed the statistical analysis. SC, RB, MC, GL, and CB-D contributed to the critical interpretation of the data and revision of the manuscript. All authors reviewed the manuscript.

## Funding

The authors acknowledge the generous support from the British Heart Foundation and the Cardiovascular Theme of the NIHR Bristol Biomedical Research Centre. The funders played no role in the design of the study, in the collection, analysis, and interpretation of data, or in the decision to submit the manuscript for publication.

## Conflict of Interest

CB-D is the CEO (part-time) of the Society for Cardiovascular Magnetic Resonance, and she received speaker's fees from Circle Cardiovascular Imaging and Siemens. The remaining authors declare that the research was conducted in the absence of any commercial or financial relationships that could be construed as a potential conflict of interest.

## Publisher's Note

All claims expressed in this article are solely those of the authors and do not necessarily represent those of their affiliated organizations, or those of the publisher, the editors and the reviewers. Any product that may be evaluated in this article, or claim that may be made by its manufacturer, is not guaranteed or endorsed by the publisher.
